# A Surgical Technique for Tibial Tubercle Avulsion Fractures Using Transpatellar Suture Tape Tension Band and De-tensioning Suture Anchors

**DOI:** 10.1016/j.eats.2024.103116

**Published:** 2024-07-14

**Authors:** Francesco Bosco, Alessandro Ghirri, Domenico Lewis Battaglia, Fortunato Giustra, Marcello Capella, Alessandro Massè

**Affiliations:** a Department of Precision Medicine in Medical, Surgical and Critical Care, University of Palermo, Palermo, Italy; b Department of Orthopedic and Traumatology (DICHIRONS), University of Palermo, Palermo, Italy; c Department of Orthopaedics and Traumatology, G.F. Ingrassia Hospital Unit, Azienda Sanitaria Provinciale 6, Palermo, Italy; d Department of Orthopaedics and Traumatology, Ospedale San Giovanni Bosco–Azienda Sanitaria Locale Città di Torino, Turin, Italy; e Department of Orthopaedics and Traumatology, Centro Traumatologico Ortopedico, University of Turin, Turin, Italy

## Abstract

This article aims to present a comprehensive technical note detailing our preferred treatment approach for tibial tuberosity avulsion fractures in the adult and elderly populations, particularly in scenarios characterized by low tissue quality and limited bone stock. Existing literature on this fracture type is scarce, with many described techniques relying on optimal bone quality for effective screw fixation of the tibial tuberosity. Various methods for tibial tuberosity avulsion fixation include K-wires, cannulated screws, staples, tension bands, suture anchors, and in select cases, direct transosseous sutures. Our technique focuses on robustly supporting the extensor mechanism through a synergistic combination of de-tensioning suture anchors, tension band suture taping, and suture augmentation of the patellar tendon. This approach addresses the challenges posed by compromised tissue quality and limited bone stock, offering a valuable contribution to the management of these fractures.

Extensor apparatus injuries consist of acute or chronic quadriceps and patellar tendon ruptures, patellar fractures, and tibial tuberosity avulsion fractures (TTAFs). Chronic ruptures are challenging because of the extensor mechanism’s proximal retraction, which may require allograft use, transosseous traction, or a 2-stage surgical procedure.^1^ However, acute injuries to the knee extensor mechanism are often complicated by local edema, bleeding, and possible lack of soft tissue or cutaneous integrity.^1^

The common biomechanical cause of the aforementioned injuries is a sudden quadriceps contraction leading to a tensile overload of the extensor mechanism.^2^, ^3^, ^4^ The fracture pattern is even less common in adults, and very few cases have been reported in the literature.^5^^,^^6^ Surgical treatment aims to restore the extensor mechanism’s integrity and function. Because this fracture pattern is not usually encountered in daily clinical practice, very few articles have been published in recent years.^5^^,^^6^ Described surgical treatments for TTAFs currently consist of patellar tendon de-functioning anchors, screw fixation of the tibial tuberosity (TT), and tension band wiring.

This article describes our technique of choice for TTAFs. The advantages of this technique that support our surgical choice are strong fixation of the TT, which can be adopted in the elderly population with low bone quality; a de-tensioning mechanism of the patellar tendon without complete loss of function of the tendon; and the use of high-resistance tape with lower reported complication rates than cerclage wires.^7^

## Surgical Technique

The surgical technique is presented in Video 1.

### Preoperative Assessment

The patient is evaluated preoperatively with a clinical examination and standard knee radiographs. Complete loss of function of the extensor apparatus is often a sign of an extensor mechanism injury. Proximal migration of the patella or avulsion fracture is easily diagnosed using standard knee radiographs. In the case of tibial avulsion fracture, a computed tomography scan is not mandatory. Still, it should be considered in cases of femoral-tibial joint involvement or comminuted fracture of the TT.^8^

### Patient Positioning and Anesthesia

The procedure is conducted with the patient under spinal or general anesthesia. The patient is placed in the supine decubitus position with a pneumatic tourniquet applied around the proximal thigh of the affected limb. The C-arm machine is positioned contralateral to the surgeon, and the operating table is shifted away from the base to allow complete access to the C-arm. The surgeon evaluates the quality of the anteroposterior and lateral knee radiographs preoperatively to ensure correct visualization throughout the surgical procedure.

### Surgical Approach

**Fig 1:**
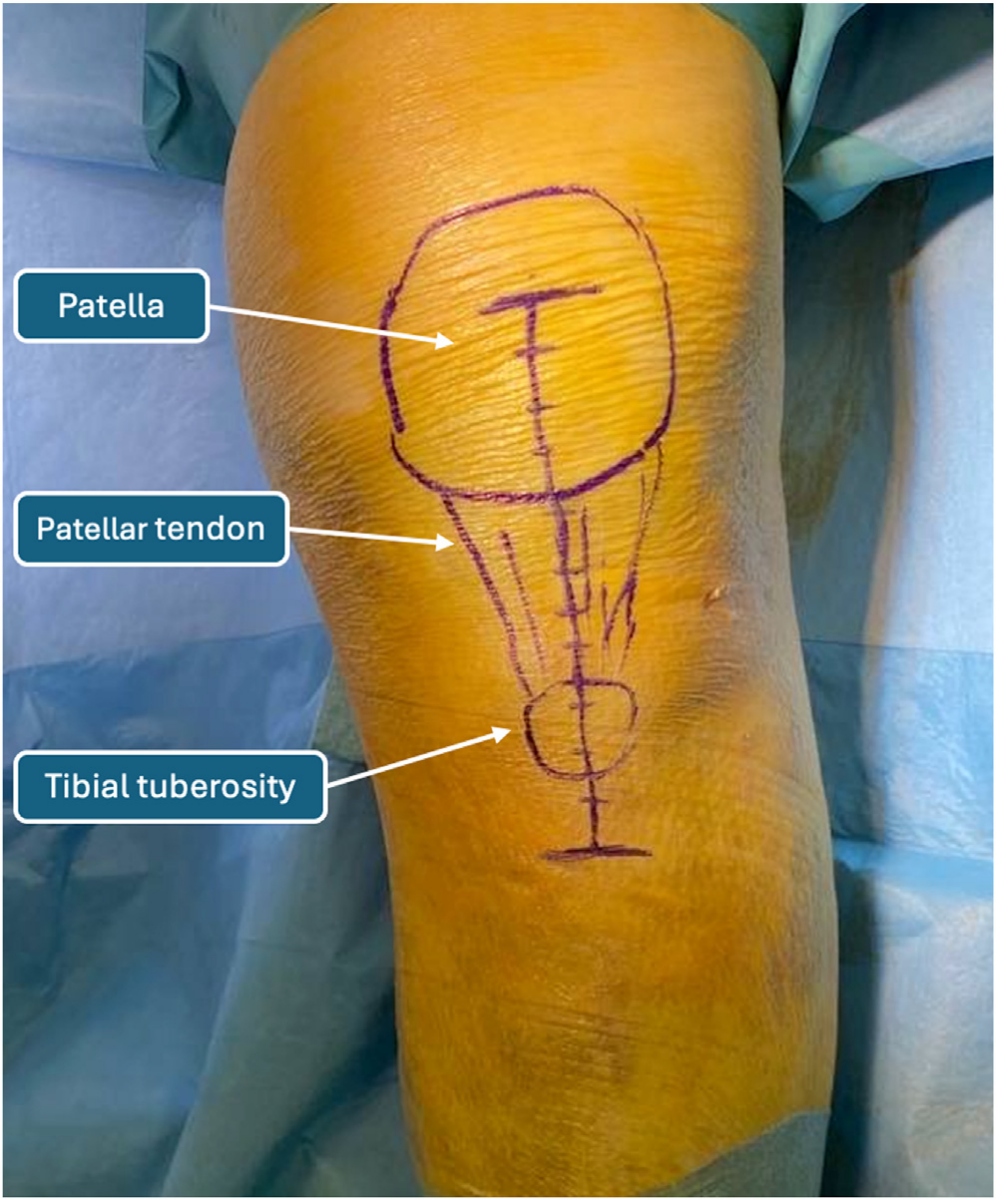
Anatomic landmarks.

**Fig 2:**
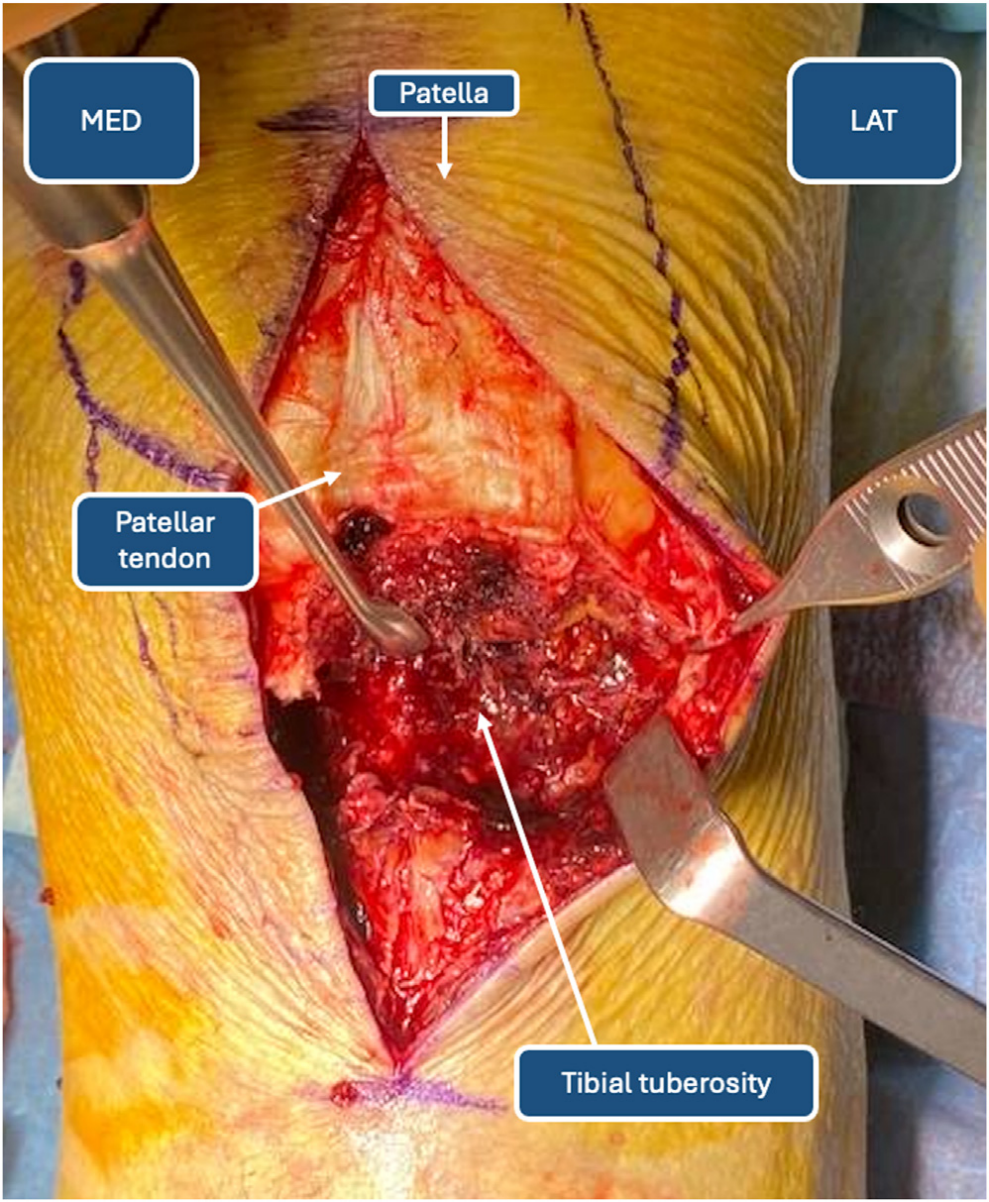
Tibial tuberosity and patellar tendon identification. (LAT, lateral side; MED, medial side.)

The patella is identified by palpating its borders, and by use of a dermographic pen, the contour is drawn for reference. The TT is then identified and marked with the sterile pen, ensuring a valuable reference point for determining the patellar tendon’s location (Fig 1). The surgeon should bear in mind the proximal migration of the patella during landmark identification because it is not found in its native position. The skin incision is performed from the center of the patella, exposing the lower half after dissection, but later reduction of the fracture will allow complete visualization of the patella. The skin incision should be extended 4 cm distally to the TT, allowing correct reduction and fixation of the TT to its anatomic position. During initial dissection, hematomas are removed and thorough surgical irrigation is performed to isolate the TT accurately (Fig 2).

### Patellar Tendon Preparation

**Fig 3:**
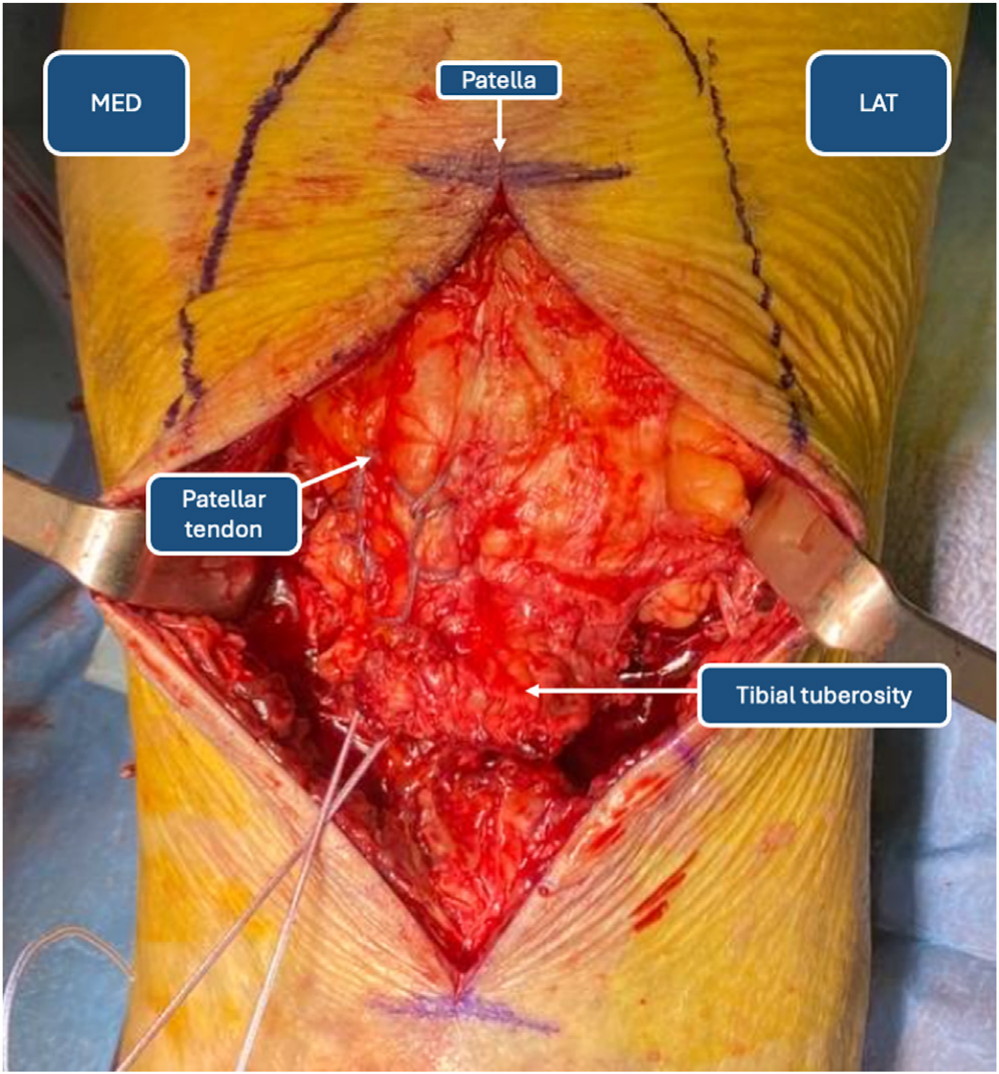
Krackow suture reinforcement of patellar tendon. (LAT, lateral side; MED, medial side.)

**Fig 4:**
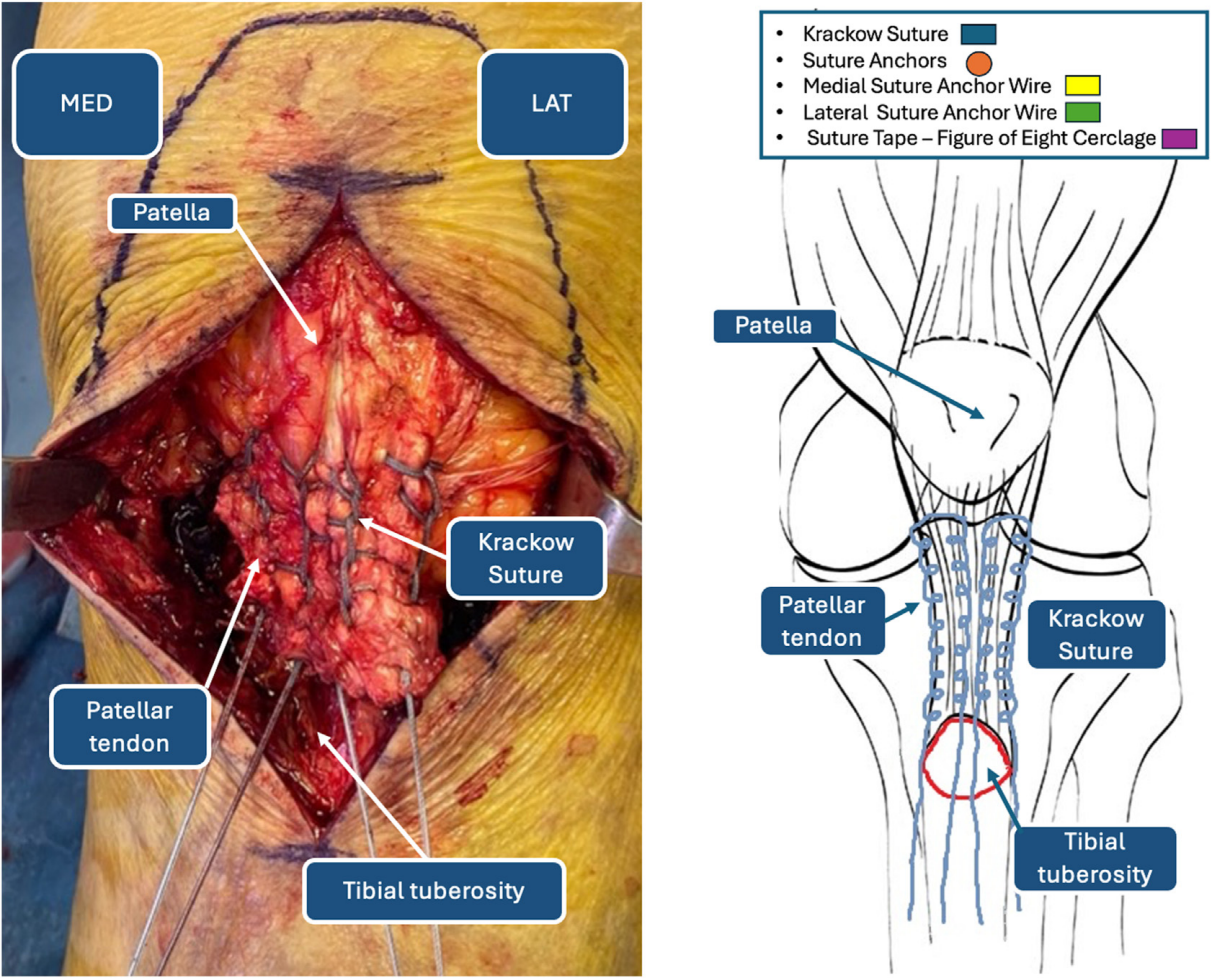
Double-row Krackow reinforcement of patellar tendon: intraoperative image (left) and iconographic representation (right). (LAT, lateral side; MED, medial side.)

The patellar tendon is identified through blunt dissection of the soft tissues anteriorly. Accurate isolation of the peritenoneum should be performed to ensure better healing, although post-traumatic lesions may irreversibly damage the tendon sheath. The tendon is reinforced medially with No. 5 FiberWire (Arthrex, Naples, FL) distally to proximally and distally again using a Krackow suture technique (Fig 3). The procedure is repeated on the lateral border of the patellar tendon. At the end of tendon preparation, 2 couples of wires should be available at the distal end of the tendon (Fig 4).

Double reinforcement of the patellar tendon with the Krackow technique is fundamental in the case of low tissue quality in elderly patients. The high-resistance characteristics of FiberWire, sustained by its multi-strand construct of ultrahigh-molecular-weight polyethylene, ensure a long-lasting construct.

### Temporary Fracture Reduction and Tibial Tunnel Creation

**Fig 5:**
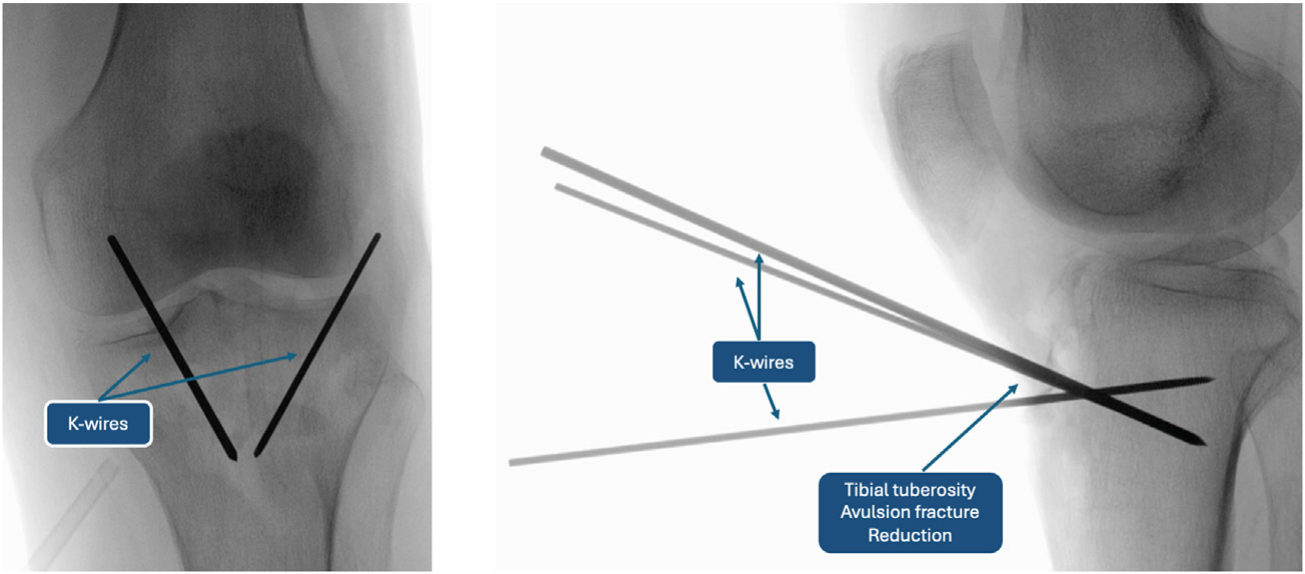
Temporary fixation of tibial tuberosity with K-wires: anteroposterior (left) and lateral (right) knee radiographs.

**Fig 6:**
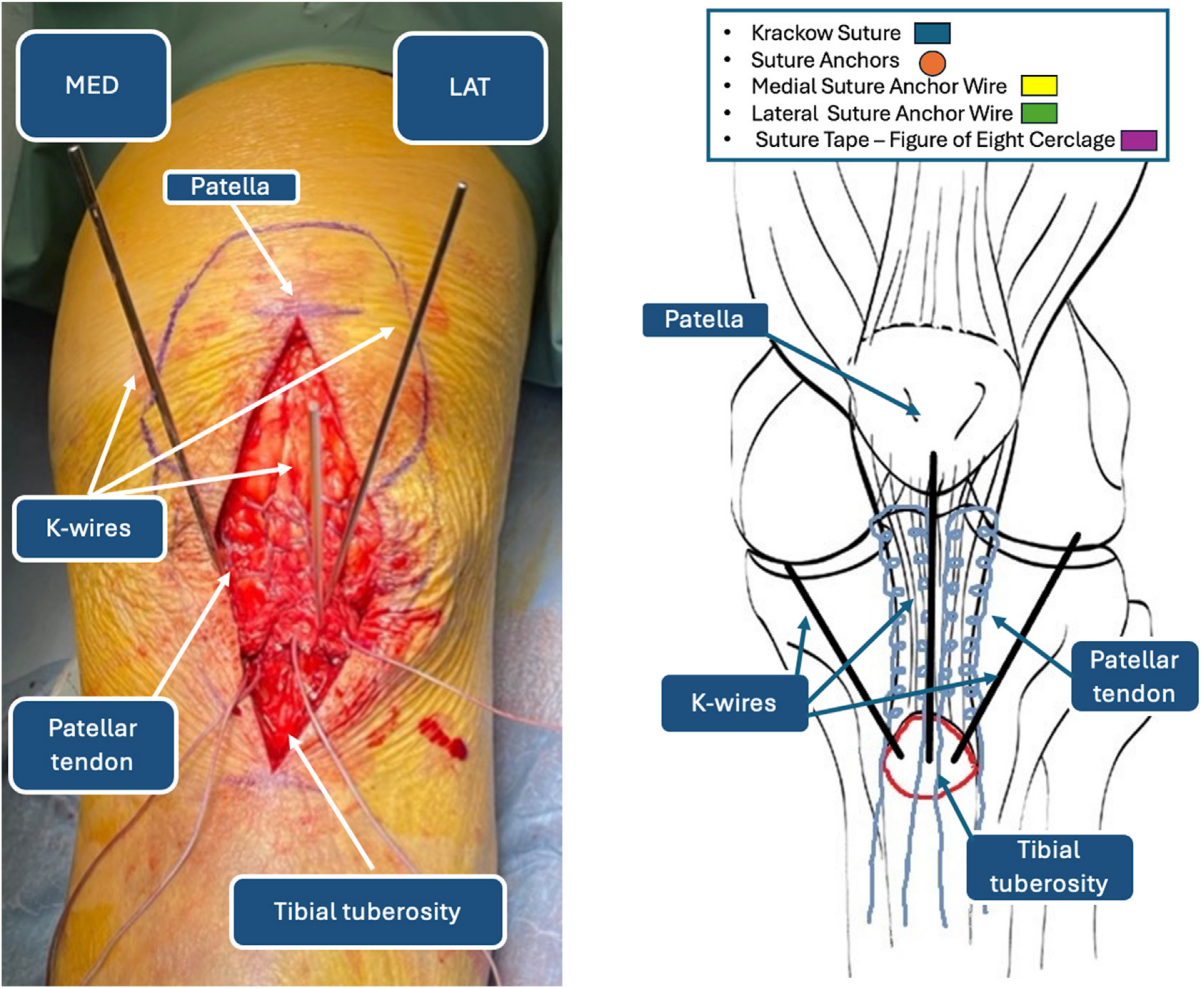
Temporary fixation of tibial tuberosity with K-wires: intraoperative image (left) and iconographic representation (right). (LAT, lateral side; MED, medial side.)

**Fig 7:**
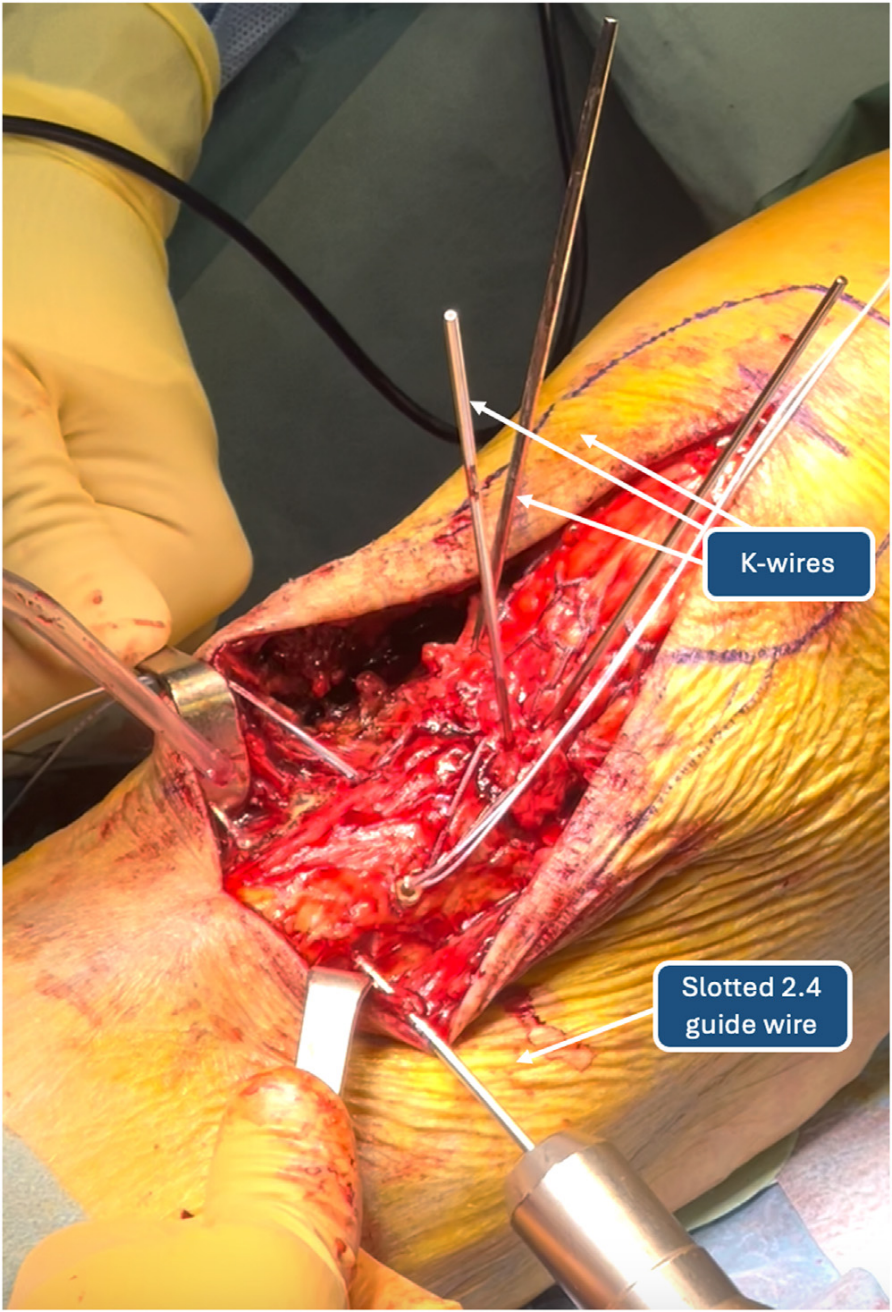
Drilling of tibial tunnel with 2.4-mm guidewire.

The surgeon proceeds with reduction of the avulsion fracture with Backhaus forceps and temporary fixation with 1.2-mm Kirschner wires (Figs 5 and 6). Correct fracture reduction is evaluated through intraoperative radiographic images, so complete visualization of the patella is achieved through distal migration of the latter. Subsequently, a point 4 cm distal and 1 cm lateral is identified on the lateral tibial cortex. A slotted 2.4-mm guidewire creates a transosseous tibial tunnel distal to the TT and parallel to the tibial-femoral joint line. As a helpful tip, we recommend leaving the 2.4-mm guidewire as a reference inside the tibial tunnel until the next surgical step (Fig 7).

### Fixation of De-tensioning Suture Anchors

**Fig 8:**
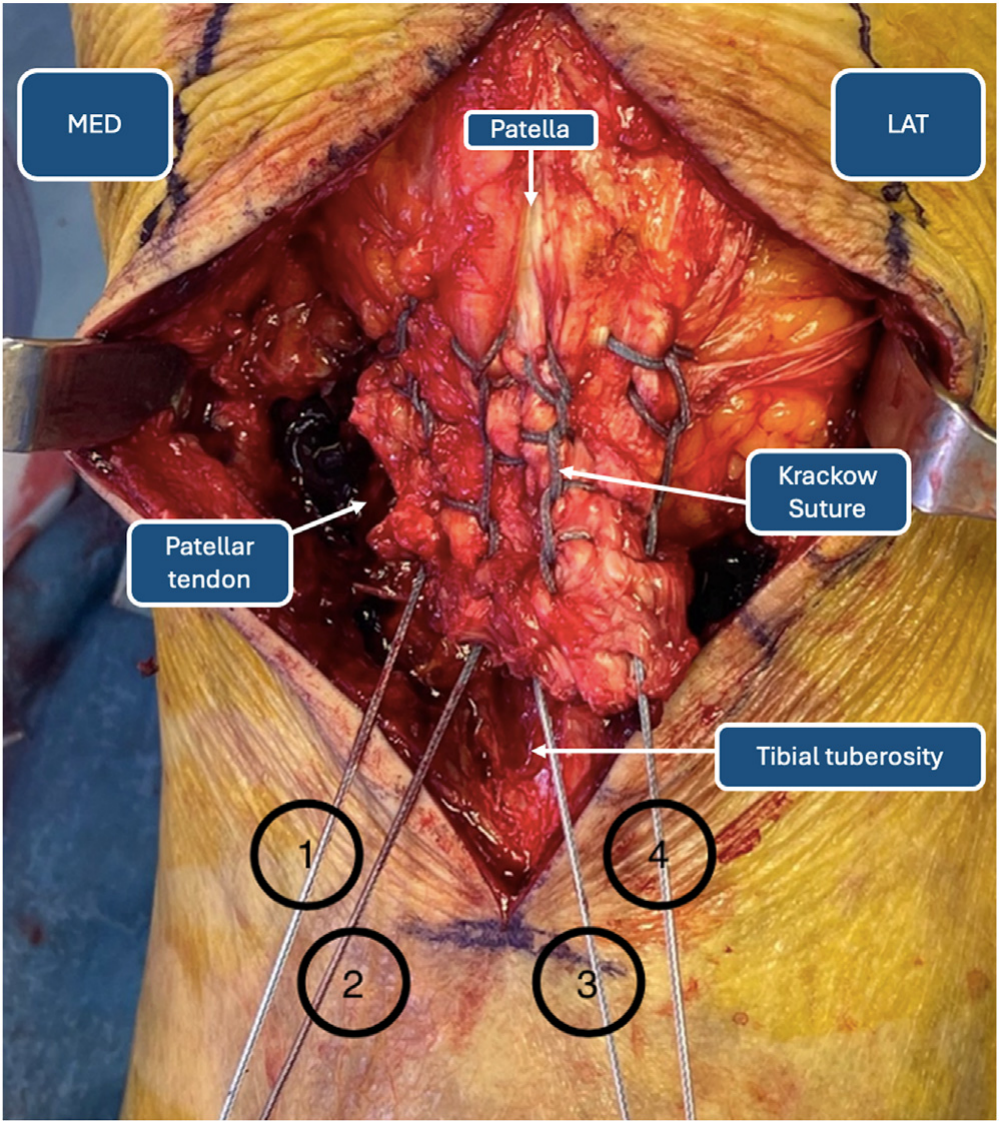
Distal ends of FiberWire sutures. (LAT, lateral side; MED, medial side.)

**Fig 9:**
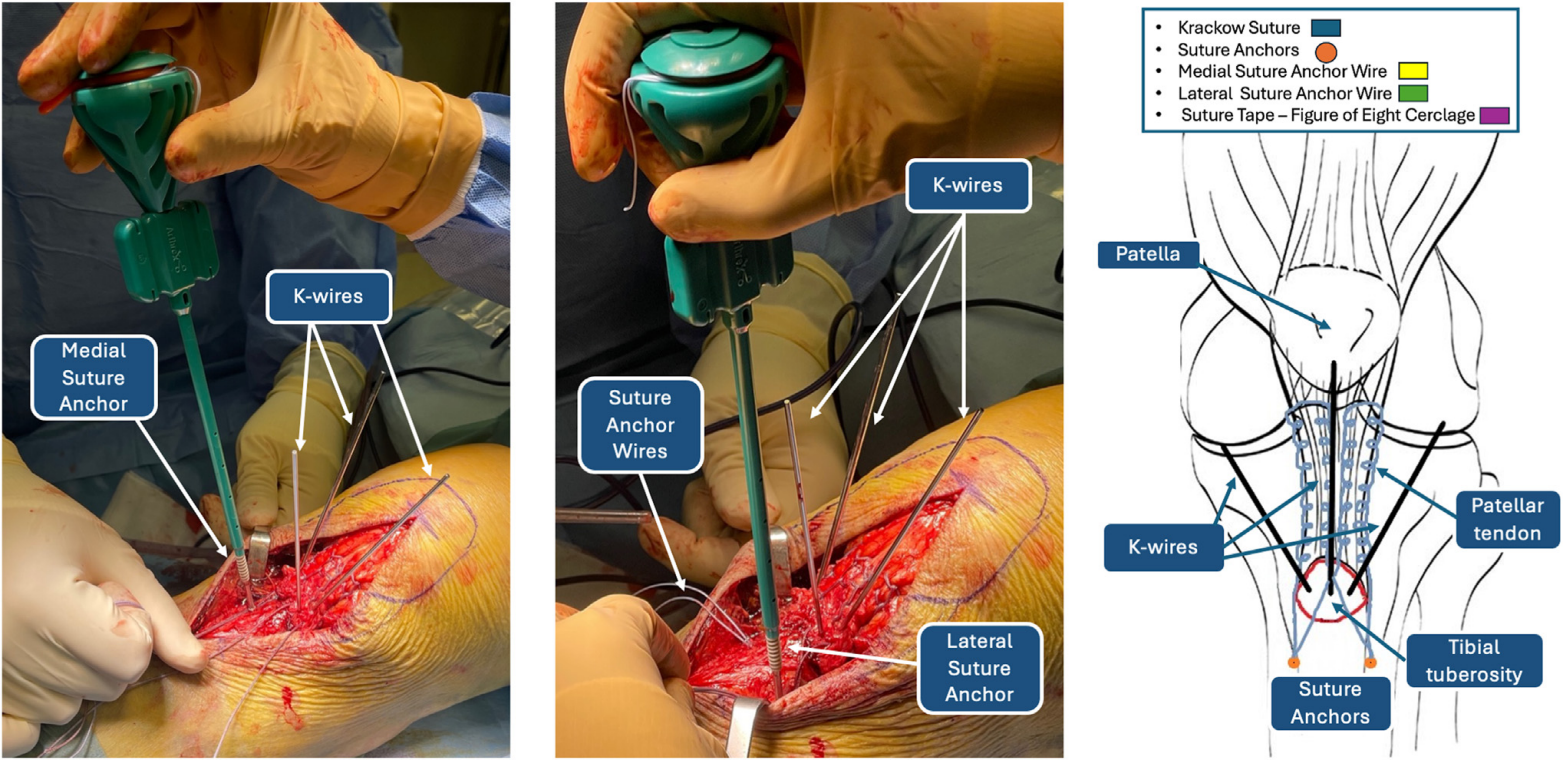
FiberWire tibial fixation using SwiveLock anchor. FiberWires 1, 2, and 3 are fixated distally to the tibial tuberosity through a SwiveLock knotless anchor (left). FiberWires 2, 3, and 4 are fixated distally to the tibial tuberosity through a SwiveLock anchor (middle). Iconographic representation (right).

**Fig 10:**
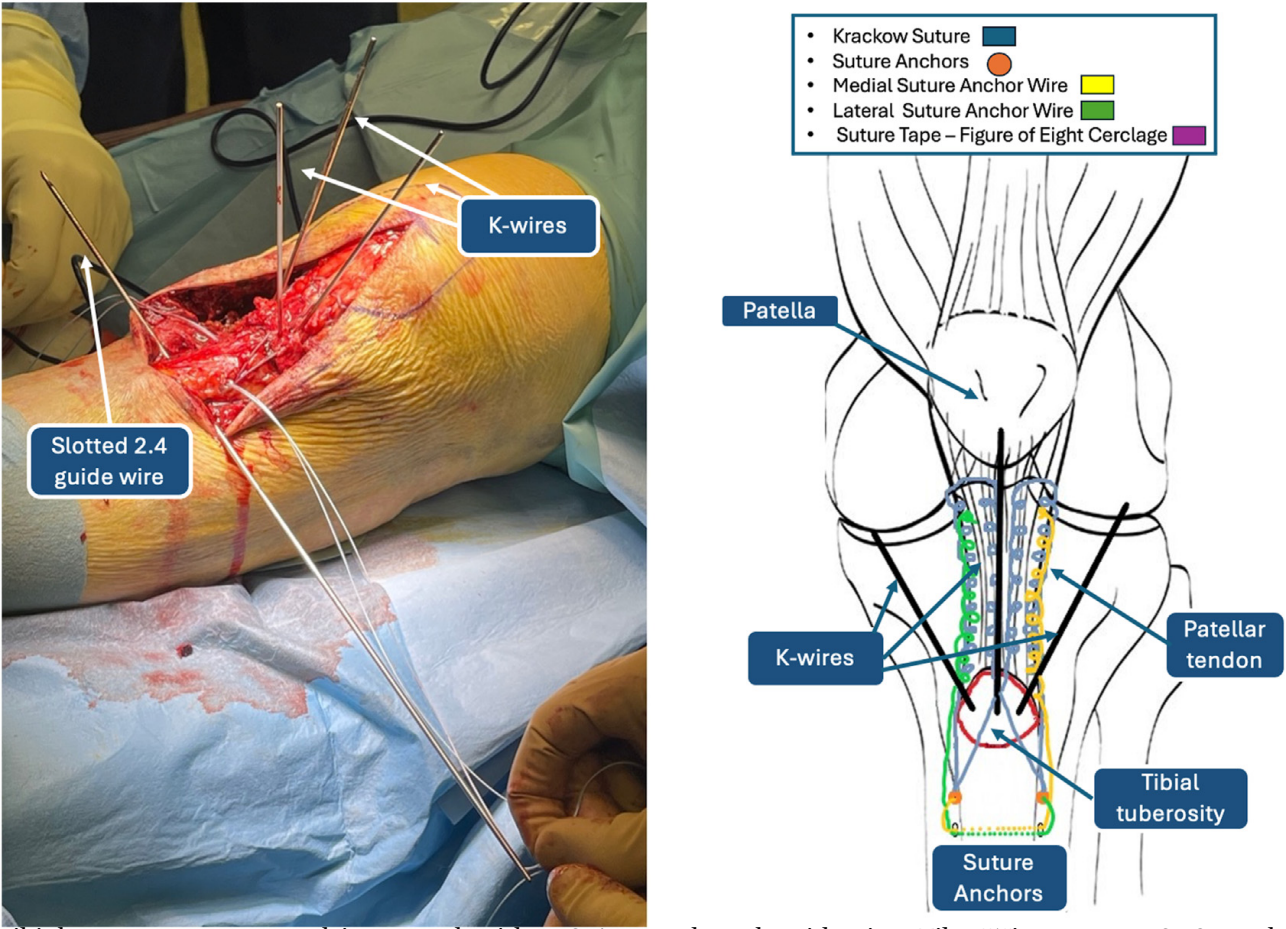
The tibial transosseous tunnel is created with a 2.4-mm slotted guidewire. FiberWire sutures 2, 3, and 4 are passed through the tibial tunnel with a shuttle suture.

As shown in Figure 8, the distal ends of the FiberWire sutures used to reinforce the patellar tendon have been numbered 1 through 4. Wires 2 and 3 are crossed in a contralateral manner on the TT. This procedure step will result in wires 1, 2, and 3 on the medial aspect and wires 2, 3, and 4 on the lateral side. Both couples of wires are then inserted into a fully threaded SwiveLock knotless anchor (Arthrex). The SwiveLock anchors are fixated distally to the TT. Fixation of the SwiveLock knotless anchors is achieved through 2 holes, drilled with a 4.5-mm noncannulated drill, 2 cm distally to the TT on the lateral and medial tibial cortex (Fig 9). The distal ends of lateral wires 2, 3, and 4 are inserted in the slotted 2.4-mm guidewire and passed through the tibial tunnel, together with an additional No. 2 nylon shuttle suture, to the medial side (Fig 10). The shuttle suture then passes FiberWires 1, 2, and 3 to the lateral side. In addition, a FiberTape (Arthrex) is prepared for the next surgical step and passed together with the FiberWires through the transosseous tibial tunnel. The free ends of the FiberWires that have been passed through the tibial tunnel are then sutured to the patellar tendon through a threadless surgical needle.

### High-Resistance Suture Tape Patellar Tendon Cerclage

**Fig 11:**
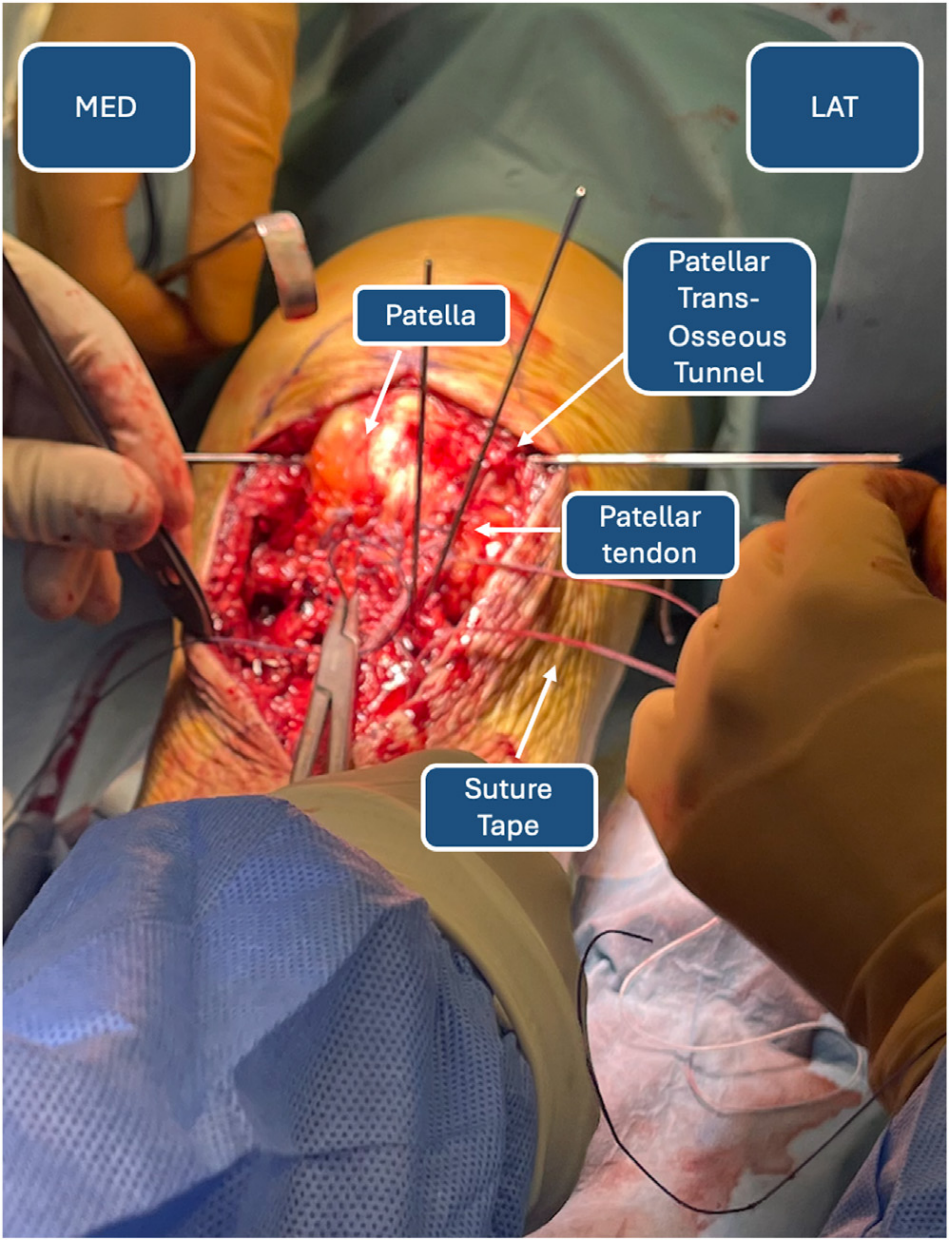
Patellar transosseous tunnel. (LAT, lateral side; MED, medial side.)

**Fig 12:**
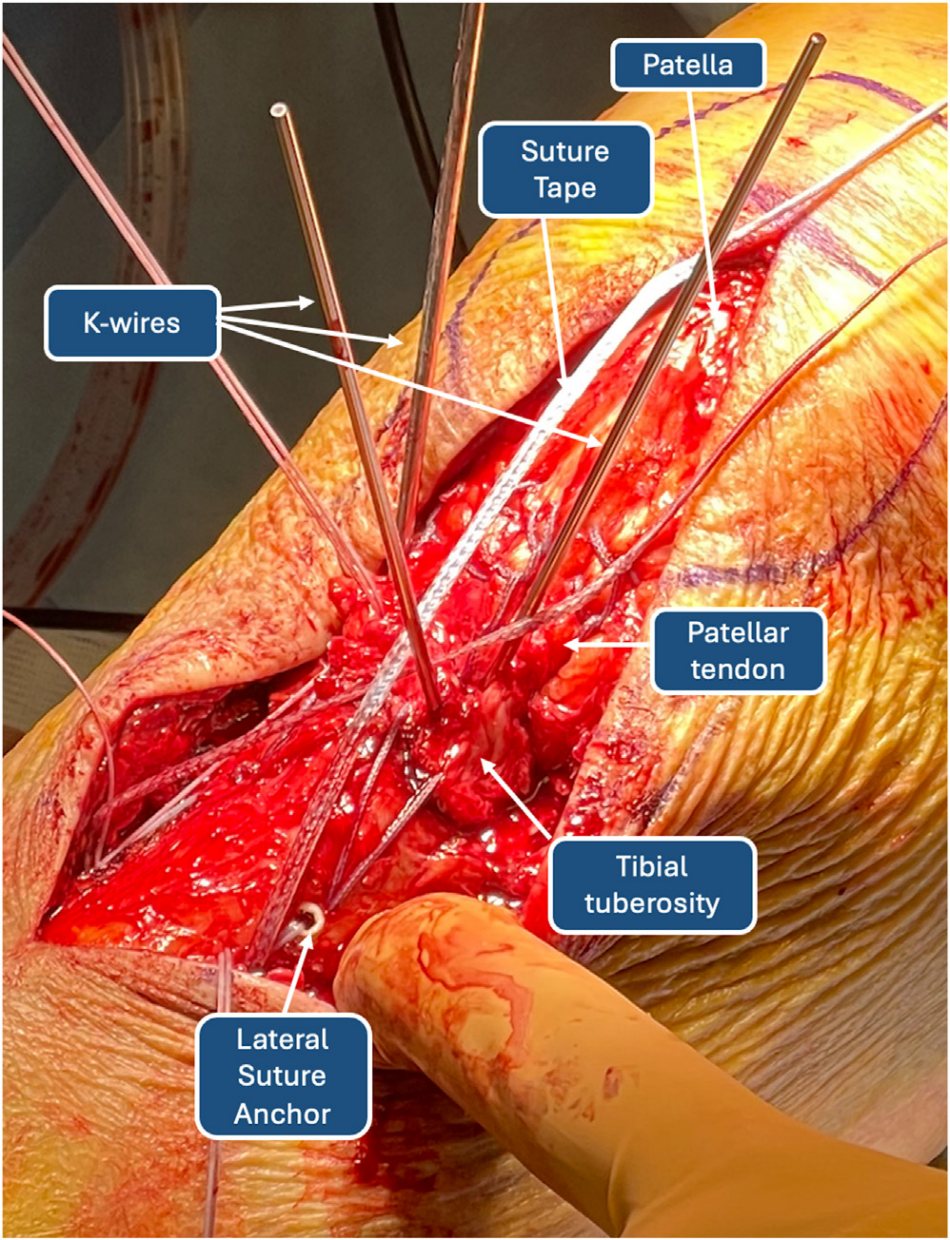
FiberTape figure-of-8 tension band–suture construct for de-tensioning mechanism of patellar tendon.

**Fig 13:**
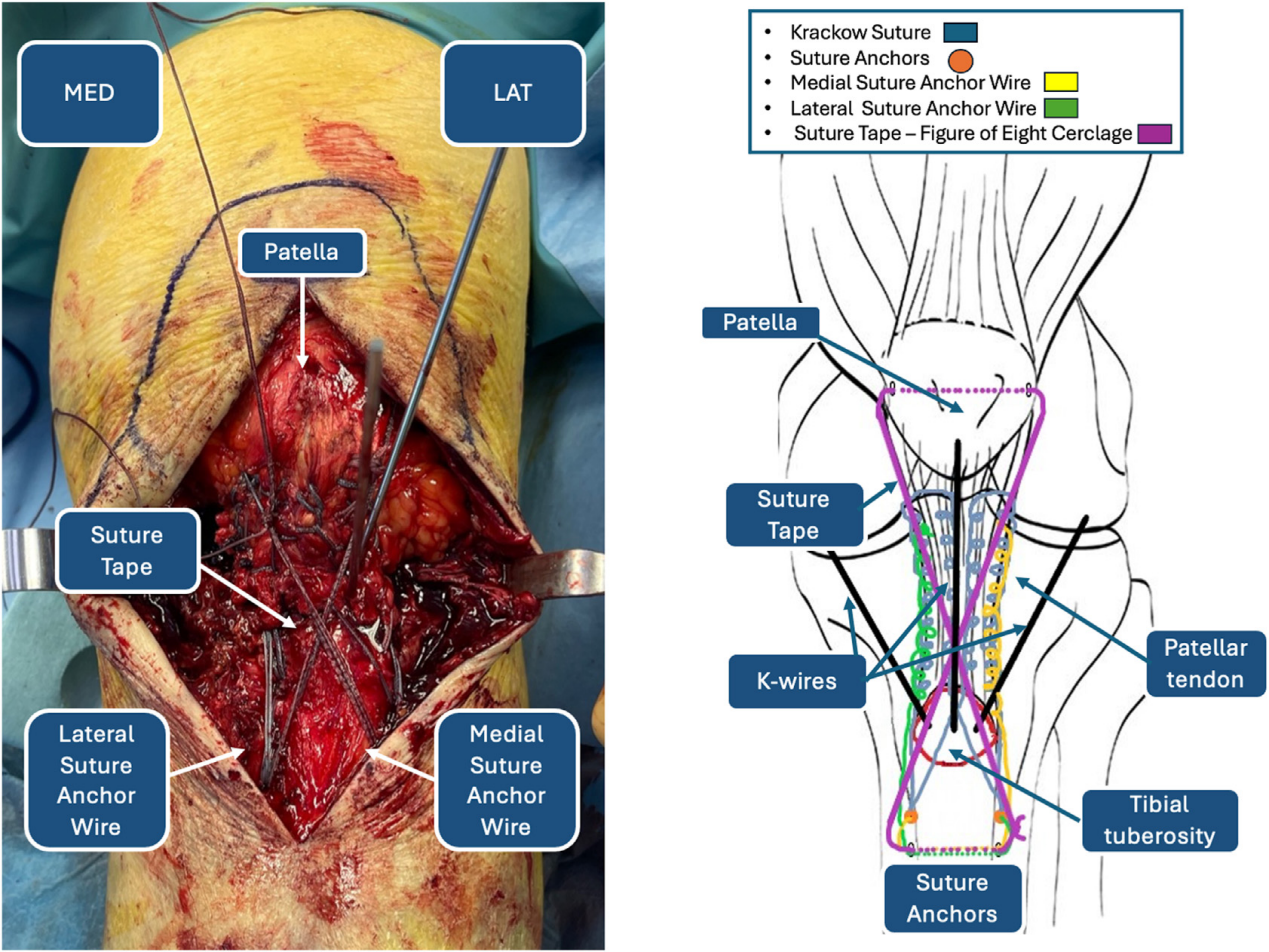
Final construct. (LAT, lateral side; MED, medial side.)

Once distal migration of the patella is achieved, a transverse patellar tunnel is drilled with the slotted 2.4-mm guidewire (Fig 11). The correct positioning of the tunnel is verified with the C-arm machine. The FiberTape that had previously been passed through the transosseous tibial tunnel is then crossed on the TT and inserted in the guidewire. The construct created is a well-known figure-of-8 cerclage of the patellar tendon (Fig 12). The cerclage is tensioned according to surgeon preference. Then, the stability of the sutures is verified through gentle flexion-extension movements of the knee, and the K-wires are removed from the TT. The final construct is represented in Figure 13. An additional cannulated screw on the TT might be considered in single-fragment fractures in patients with good bone quality (Table 1).

**Table 1:** Highlights of Surgical Technique

Perform an incision from the center of the patella, and extend the skin incision distally to the tibial tuberosity. During the initial dissection, remove hematomas, thoroughly irrigate the area, and isolate the tibial tuberosity for further evaluation and preparation.
Identify and isolate the patellar tendon. Once isolated, reinforce the tendon using FiberWire and the Krackow suture technique.
Reduce the avulsion fracture, and provide temporary fixation with Kirschner wires. Simultaneously, create a tibial tunnel distal to the tibial tuberosity to facilitate subsequent steps in the surgical procedure.
Use SwiveLock knotless anchors for fixation, inserting the distal ends of FiberWire sutures. Cross the wires in a contralateral manner on the tibial tuberosity, and pass them through the tibial tunnel using a nylon shuttle suture.
Drill a transverse patellar tunnel with a guidewire; then, cross FiberTape on the tibial tuberosity to form a figure-of-8 cerclage. Tension the cerclage appropriately, verify stability through gentle knee flexion-extension movements, and conclude the procedure by removing the Kirschner wires.

### Postoperative Protocol

We suggest 4 weeks of cast positioning with the knee in full extension, followed by 2 weeks in a genu brace with a 0° extension and 30° flexion block. Physiotherapy at this stage may be considered for early passive range-of-motion exercises. Weight bearing with a full-extension genu brace is permitted at 30 days postoperatively, followed by active range-of-motion exercises, progressively gaining 30° of flexion weekly. Full flexion should be achieved at 3 months postoperatively. At 60 days postoperatively, progressive free weight bearing may be allowed if postoperative radiographs show acceptable fracture healing signs.

## Discussion

TTAFs are rare occurrences in the adult population, resulting in a notable scarcity of scientific literature addressing their treatment and outcomes. These fractures typically arise from sudden eccentric contractions of the quadriceps, primarily affecting the proximal extensor mechanism apparatus. However, alternative mechanisms such as hyperflexion or direct trauma can also lead to TTAFs.^9^^,^^10^

Various methods are available for fixing TTAFs, including K-wires, cannulated screws, staples, tension bands, suture anchors, and in certain scenarios, direct transosseous sutures.^2^^,^^3^^,^^10^, ^11^, ^12^, ^13^, ^14^, ^15^ Our technique involves the use of transpatellar suture tape tension band in conjunction with de-tensioning suture anchors, ensuring robust reinforcement. There is consensus regarding the significance of augmentation or internal bracing techniques, particularly in cases of compromised tissue quality.^11^, ^12^, ^13^, ^14^, ^15^, ^16^ Table 2 shows a comprehensive overview of pearls and potential pitfalls of the described surgical technique.

**Table 2:** Pearls and Pitfalls

Pearls
The surgeon should consider the proximal migration of the patella during landmark identification because the patella is not found in its native position owing to the patellar tendon lesion.
Double reinforcement of the patellar tendon with the Krackow technique is fundamental in the case of low tissue quality in elderly patients.
A slotted 2.4-mm guidewire creates a transosseous tibial tunnel distal to the tibial tuberosity and parallel to the tibial-femoral joint line. The 2.4-mm guidewire is left inside the tibial tunnel as a reference until the next surgical step.
The surgeon should verify correct fracture reduction with the C-arm machine and assess the stability of the construct with careful flexion-extension movements.
Pitfalls
Relying solely on clinical examination findings and standard knee radiographs may lead to lack of a comprehensive evaluation. Failure to consider sonography for early detection of soft-tissue injuries or to assess multiligamentous injuries with magnetic resonance imaging may result in overlooked issues.
Insufficient blunt dissection and isolation of the peritenoneum may result in compromised healing. Overlooking the need for double reinforcement in cases of low tissue quality, particularly in elderly patients, may compromise the long-term stability of the construct.
Errors in achieving correct reduction of the avulsion fracture and correct positioning of the tibial tunnel may lead to improper alignment and fixation of the tibial tuberosity, impacting the overall stability of the construct.
Inaccurate tensioning of the FiberTape during creation of the figure-of-8 cerclage may result in inadequate stability. Failure to verify stability through knee movements can lead to undetected issues in the suture tape patellar tendon cerclage.

In conclusion, in our technique, transpatellar suture tape tension are used with de-tensioning suture anchors, ensuring robust reinforcement of the tendon’s insertion point. Applying high-resistance suture tape in a figure-of-8 cerclage pattern around the patellar tendon, coupled with a transverse patellar transosseous tunnel, facilitates a de-tensioning configuration for the tendon. This configuration is particularly advantageous in cases of low-quality bone stock because it promotes an enhanced healing process by alleviating tension on the tendon.

## Disclosures

All authors (F.B., A.G., D.L.B., F.G., M.C., A.M.) declare that they have no known competing financial interests or personal relationships that could have appeared to influence the work reported in this paper.

## Supplementary Data

Video 1 Surgical technique for treating tibial tubercle avulsion fractures. Our technique consists of a combined mechanism of de-tensioning suture anchors and high-resistance suture tape acting as a tension band fixated through 2 transosseous tunnels. This is a rare fracture pattern in the elderly population in opposition to adolescents, in whom it is more frequently encountered. The patient is placed in the supine decubitus position with a pneumatic tourniquet. The main anatomic landmarks are identified and marked with a dermographic pen. The skin incision is performed from the center of the patella and extended 4 cm distal to the tibial tuberosity. Thorough surgical dissection is performed to isolate the tibial tuberosity accurately. The patellar tendon is reinforced with high-resistance wires through a Krackow suture medially and laterally. At the end of this procedure, 2 couples of wires should be available distally on the patellar tendon. The tibial tuberosity is then fixated with 2 or 3 K-wires. The medial ends of the wires are passed in a contralateral manner and inserted into a knotless suture anchor. Fixation of the knotless anchors is achieved through 2 holes drilled 2 cm distally to the tibial tuberosity on the lateral and medial tibial cortex. A slotted 2.4-mm guidewire is used to create a transosseous tibial tunnel distally to the tibial tuberosity. The distal ends of the lateral wires are inserted into the guidewire and passed through the tibial tunnel together with an additional suture shuttle and high-resistance tape to the medial side. The suture shuttle is then used to pass the medial FiberWires to the lateral side. The free ends of the wires that have been passed through the tibial tunnel are sutured to the patellar tendon through a threadless surgical needle. A transpatellar tunnel is performed through a 2.4-mm slotted guidewire. The tape that has been passed through the transosseous tibial tunnel is then crossed on the tibial tuberosity and inserted into the slotted guidewire. The construct created is a figure-of-8 cerclage of the patellar tendon.
